# Inhibition of muscle fibrosis results in increases in both utrophin levels and the number of revertant myofibers in Duchenne muscular dystrophy

**DOI:** 10.18632/oncotarget.4021

**Published:** 2015-05-21

**Authors:** Oshrat Levi, Olga Genin, Corrado Angelini, Orna Halevy, Mark Pines

**Affiliations:** ^1^ Institute of Animal Sciences, Volcani Center, Bet Dagan, Israel; ^2^ Department of Neurosciences, University of Padova, and IRCCS S.Camillo, Lido, Venice, Italy; ^3^ Department of Animal Sciences, the Hebrew University of Jerusalem, Rehovot, Israel

**Keywords:** fibrosis, muscular dystrophy, utrophin, collagen, halofuginone

## Abstract

Duchenne Muscular Dystrophy is characterized by: near absence of dystrophin in skeletal muscles; low percentage of revertant myofibers; up-regulation of utrophin synthesis; and a high degree of muscle fibrosis. In patient quadriceps femoris biopsies (*n* = 6, ages between 3–9 years) an inverse correlation was observed between the levels of collagen type I – representing fibrosis – and the levels of utrophin. This correlation was independent of the patient's age and was observed in the entire muscle biopsy sections. In the *mdx* mice diaphragm (*n* = 6/group), inhibition of fibrosis by halofuginone resulted in increases in the levels of utrophin. The utrophin/fibrosis relationships were not limited to collagen type I, but also applied to other constituents of the fibrosis machinery. The inverse correlation was found also in old *mdx* mice with established fibrosis. In addition, inhibition of collagen type I levels was associated with increases in the numbers of revertant myofibers, both as single myofibers and in clusters in the diaphragm and the gastrocnemius.

In summary, our results demonstrate an inverse correlation between the level of muscle fibrosis and the level of utrophin and that of the number of revertant myofibers. These findings may reveal common links between the fibrotic and utrophin-synthesis pathways and offer new insights into the regulation of utrophin synthesis.

## INTRODUCTION

The most common form of X-linked muscle dystrophy (MD) is Duchenne Muscular Dystrophy (DMD), which affects 1 in 3500 live males at birth. Progress of muscle degeneration and worsening clinical symptoms leads to death in the late teens or early twenties as a result of respiratory or cardiac failure. DMD is characterized by near absence of the protein dystrophin in skeletal muscles. The dystrophin-glycoprotein complex (DGC) connects the actin cytoskeleton of myofibers to the extracellular matrix (ECM) and is, therefore, integral to the contractile structure of muscle [1, 2]. The preliminary stage of the disease is characterized by the presence of focal groups of necrotic myofibers, muscle hypertrophy, and abnormally high levels of muscle creatine kinase (CK). In the pathological phase, repeated cycles of degeneration exhaust the regenerative capacity of muscle-specific progenitor cells (satellite cells) and fibrotic mechanisms, and thereby cause progressive replacement of the muscle tissue with collagenous connective tissue [3]. The increase in collagen deposition initiates a vicious cycle in which further muscle destruction leads to joint contractures, loss of ambulation, and death from respiratory or cardiac failure [4]. Although DMD is caused by frame-disrupting mutations in the *DMD* gene that prevent the full translation of dystrophin in muscles of DMD patients [5, 6] and in *mdx* mice – the murine model of DMD [7–9]– both exhibit sporadic low percentages of dystrophin-positive myofibers known as revertant fibers (RFs). The RFs are probably the result of clonal expansion of individual myogenic progenitors [10]; their numbers differ between different muscles at different ages but, because of their low level, they have minimal, if any effect on the clinical phenotype [11].

A promising treatment for DMD patients aims to increase levels of utrophin – the autosomal homologue of dystrophin [12]. Utrophin is a 395-kDa protein with a high degree of amino acid identity with dystrophin, and similar organizational domain structures [13–15]. Both proteins have similar affinities for binding F-actin at the amino terminus and have the ability to bind the DGC at the carboxyl terminus [16, 17]. The linkage between the two proteins and cytoskeletal actin is the main factor in resistance to deformation [18,19]. In adult skeletal muscle utrophin expression is low and limited to neuromuscular junctions [20]. Utrophin level is up-regulated in DMD patients, which indicates its ability to compensate for the lack of dystrophin but that its levels are not sufficient to prevent the disease [21]. In animal models of DMD when utrophin is over-expressed in myofibers through transgenic, viral-vector-mediated, or other means, is able to compensate functionally for the absence of dystrophin, [22–26]. Attempts have been made to up-regulate utrophin expression by means of microRNA-mediated inhibitory mechanisms [27] and by screening for compounds that can activate the utrophin promoter, and some of these compounds reached clinical trials [28–31].

The major DMD-associated complication is muscle fibrosis, characterized by increases in ECM constituents, especially collagen type I and collagen triple-helix repeat containing 1 (Cthrc1) that is involved in collagen turnover in skeletal and cardiac muscles – increases that enhance the progressive loss of muscle and its ability to function [32, 33]. In DMD and other MDs, increased fibrosis correlates with muscle destruction [3] and with respiratory and heart failure, the leading causes of death in DMD patients [34]. To elucidate possible interactions between the severity of muscle fibrosis, on the one hand, and utrophin level and the number of RFs, on the other hand, we used halofuginone, an inhibitor of Smad3 phosphorylation downstream of the TGFβ-signaling pathway [35]. In murine models of various MDs halofuginone was shown to inhibit the Smad3 phosphorylation in cardiac and skeletal muscles that was associated with decreased muscle fibrosis, and thereby to enhance motor coordination and balance [36–38]. This was achieved both in young and older *mdx* mice that exhibited established fibrosis [39] and in the myopathic hamster cardiac muscle [40]. The results of the present study suggest an inverse correlation between muscle fibrosis, on the one hand, and the level of utrophin and the number of RFs, on the other hand in DMD patients' biopsies and in *mdx* mice treated with halofuginone.

## RESULTS

### Utrophin and fibrosis in the dystrophic muscles of DMD patients

**Figure 1:**
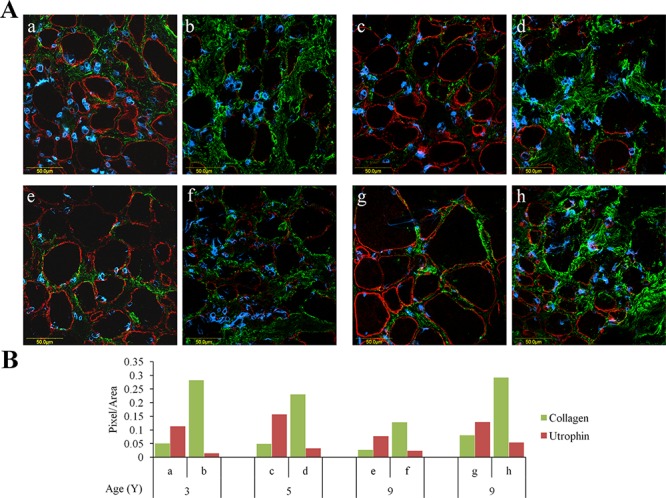
Utrophin and collagen type I levels in muscles of DMD patients QF biopsies taken from DMD patients were double-immunostained with utrophin (red) and collagen type I (green) antibodies, nuclei were stained with DAPI (blue), and all were visualized by confocal microscopy. **A.** Depiction of areas rich in either utrophin or collagen type I that were taken from four DMD patients. **B.** Image analysis quantification of utrophin and collagen type I levels presented as pixels/unit area. The levels of the collagen and utrophin signals were evaluated with NIS-Elements microscope imaging software (Nikon Instruments Inc). a, b – 3-year-old DMD patient; c, d – 5-year-old DMD patient; e, f and g, h – two 9-year-old DMD patients. Note that regardless of age, areas that are rich in collagen type I exhibit low levels of utrophin and vice versa.

**Figure 2:**
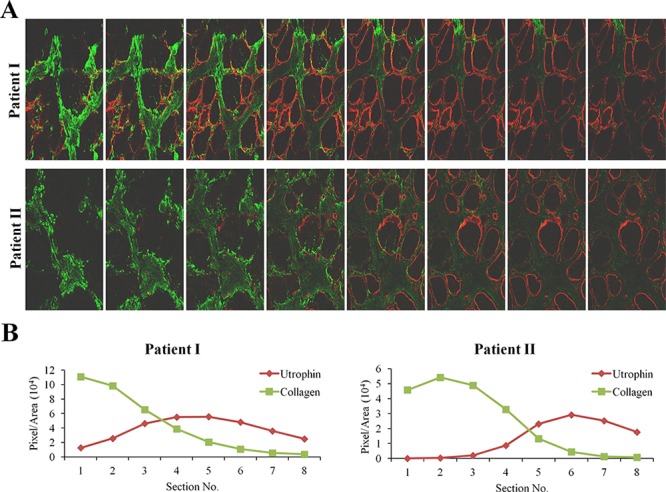
Z-stacks photo images of muscle biopsies of DMD patients **A.** QF biopsies from two patients (patient I - 4 years; patient II - 8 years) were double-immunostained with utrophin (red) and collagen type I (green) antibodies. Utrophin and collagen type I were visualized by confocal microscopy, and Z-stacks photographs (taken at 1.2 μm intervals, extending to 8-μm depth in the dystrophic muscle) were taken. **B.** Image analysis quantification of utrophin and collagen type I levels presented as pixels/unit area.

Quadriceps femoris biopsies taken from four DMD patients of different ages were double-immunostained with collagen type I and utrophin antibodies (Figure 1A). In each biopsy (performed on the entire section), irrespective of the patient's age, there were areas rich in collagen type I or utrophin; those with high levels of collagen type I displayed low levels of utrophin and vice versa (Figure 1B). In some myofibers, the whole sarcolemma was evenly stained with utrophin and in others it was unevenly or discontinuously stained. Confocal microscopy Z-stack analysis of two DMD patients' biopsies (patient 1 – age 4; patient 2 – age 8) that extended 8 μm beneath the surface of the dystrophic muscles revealed an inverse correlation between collagen type I and utrophin levels as depth into the tissue increased the level of collagen type I decreased and that of utrophin increased (Figure 2). These results were obtained in the whole muscle biopsy sections suggesting that the inverse correlation between collagen type I and utrophin prevails throughout the entire dystrophic muscle.

### Reduction in muscle fibrosis affects utrophin levels

**Figure 3:**
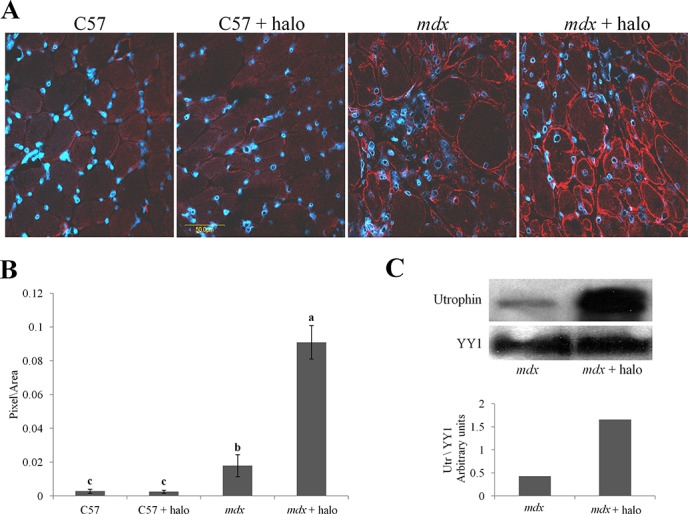
Utrophin levels in the diaphragms of *mdx* mice **A.** Mice (*mdx* or C57/Bl; *n* = 6/group) were injected IP with either halofuginone at 7.5 μg per mouse or with saline, for 10 weeks starting from the age of 4 weeks. The diaphragms were immunostained with utrophin antibodies (red) and nuclei with DAPI (blue), and visualized by confocal microscopy. **B.** Image analysis quantification of utrophin levels presented as mean pixels/unit area ± SE of the whole diaphragms of at least 6 mice/group. Means with no common superscript differ significantly (*p* < 0.05). **C.** Western blots analysis of utrophin in the *mdx* diaphragm (pool of three diaphragms in each group).

**Figure 4:**
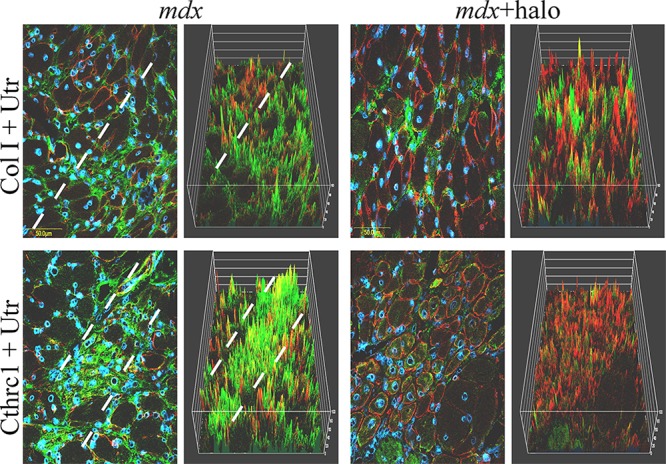
Relationship between utrophin and fibrosis Halofuginone at a concentration of 7.5 μg/mouse was injected IP into male *mdx* mice three times/week for 10 weeks, starting at 4 weeks of age. The diaphragms were double-immunostained for utrophin (red) with collagen type I (green, upper panel) or cthrc1 antibodies (green, lower panel). Nuclei were stained with DAPI (blue). In the *mdx* mice, areas with high levels of collagen (upper panel) or cthrc1 (lower panel) and low levels of utrophin were observed (separated by the dashed lines). Visualization was clearer in the 3D reconstruction. Halofuginone treatment decreased the levels of both collagen type I and cthrc1, whereas the utrophin level increased.

**Figure 5:**
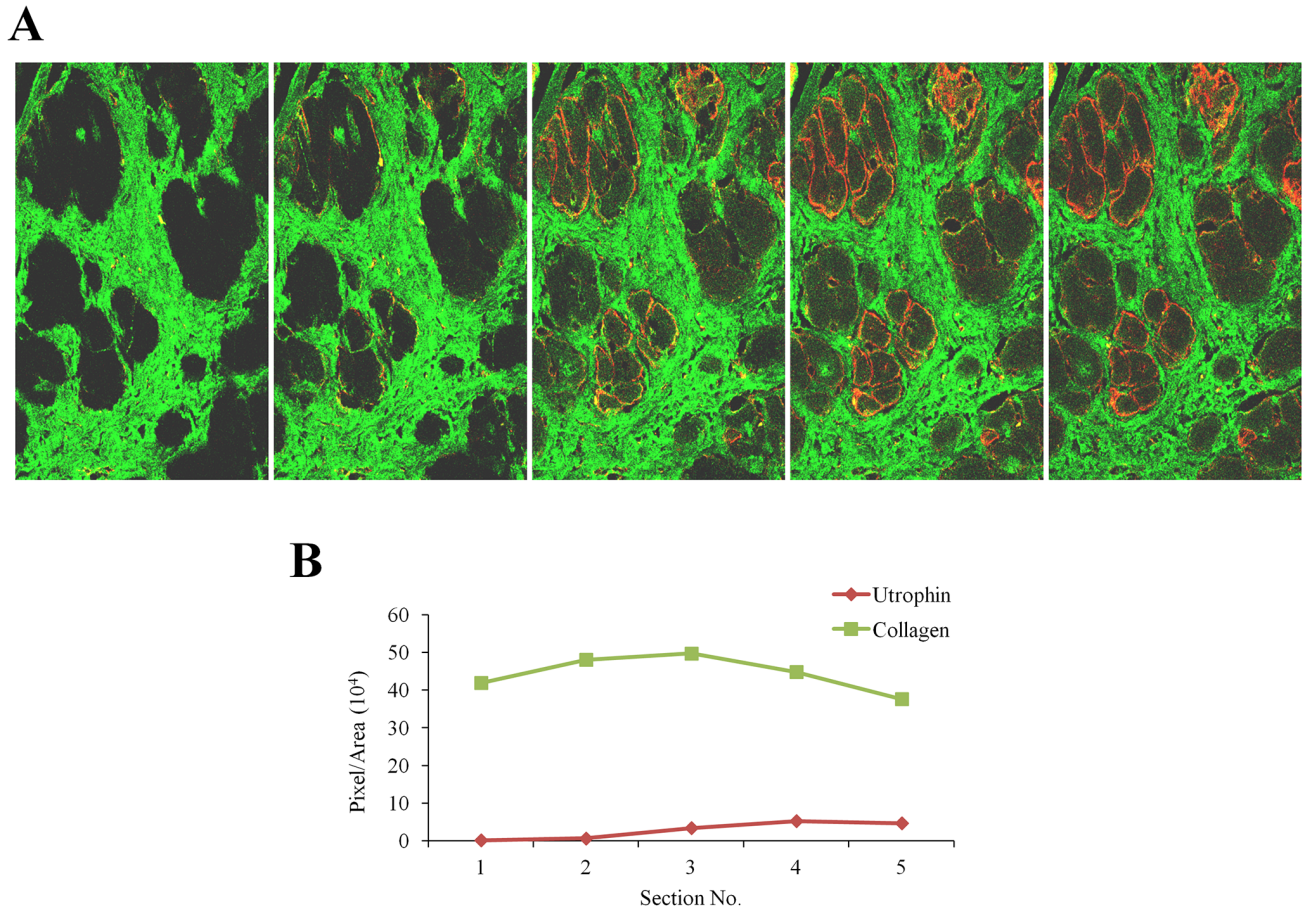
Utrophin and fibrosis in old *mdx* mice **A.** Diaphragm of 14-month-old *mdx* mouse was double-immunostained with collagen type I (green) and utrophin (red) antibodies. Utrophin and collagen type I were visualized by confocal microscopy and Z-stacks photography (taken at 1.2 μm intervals, extending to 8 - μm depth in the dystrophic muscle) were taken. **B.** Image analysis quantification of utrophin and collagen type I levels presented as pixels/unit area. At increasing depths into the tissue collagen type I levels were observed to decrease, and utrophin levels to increase.

For further investigation of the association between fibrosis and utrophin levels we used halofuginone, an inhibitor of collagen type I synthesis that serves as an anti-fibrosis therapy [38, 42]. Wild-type and *mdx* mice were treated with halofuginone at 7.5 μg/mouse, three times weekly for 10 weeks, starting from the age of 4 weeks, and control mice were injected with saline. At the end of the experiment the diaphragms were taken for utrophin immunostaining and for western blot analysis. The diaphragms of the C57/Bl mice were almost devoid of utrophin, regardless of halofuginone treatment (Figure 3A, B). Greater utrophin levels were observed in the *mdx* mice than in the C57/Bl mice, as also observed by others [43] and a further increase in utrophin levels was observed after halofuginone treatment. Image analysis of the utrophin levels in the diaphragms revealed 6 × greater levels in the *mdx* mice than in the wild-type mice and a further 5 × greater levels in the halofuginone-treated than in the untreated *mdx* mice (Figure 3B). Utrophin was expressed by both fast and slow myofibers in the diaphragm of the *mdx* mouse before and after halofuginone treatment as demonstrated by double-staining with monoclonal anti-myosin antibodies for both types of myofibers (data not shown). The halofuginone-dependent increase in diaphragm utrophin levels was confirmed by western blot analysis (Figure 3C). To evaluate any spatial association between fibrosis and utrophin, the diaphragms of the *mdx* mouse before and after halofuginone treatment were double-immunostained with anti-utrophin (red) and either anti-collagen type I (green) or anti-cthrc1 (green) antibodies (Figure 4). Both collagen type I and cthrc1 play major roles in the fibrotic reaction [33, 35, 36]. In the untreated *mdx* diaphragm the areas with reduced levels of collagen (Figure 4 upper panel) or cthrc1 (Figure 4 lower panel) were richer in utrophin-positive myofibers than the highly fibrotic areas. This was better visualized in the three-dimensional (3-D) composite images; the dashed line separates the high-collagen or -cthrc1/low-utrophin areas from the low-collagen or -cthrc1/high-utrophin areas. In the halofuginone-treated *mdx* mice both the collagen type I and cthrc1 levels were reduced and that of utrophin was increased. Furthermore, Z-stack analysis revealed very low levels of utrophin in the diaphragm of a 14-month-old *mdx* mouse that exhibited a very high level of collagen type I in the entire muscle biopsy sections (Figure 5).

### Inhibition of fibrosis resulted in increase in the number of RFs

**Figure 6:**
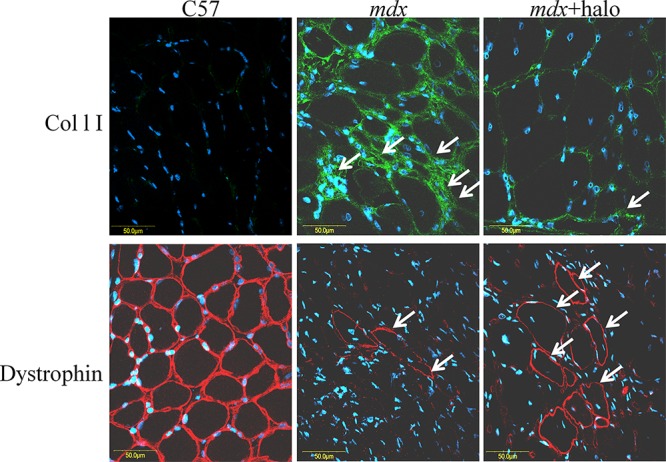
Inhibition of fibrosis and numbers of RFs Diaphragms of wild-type C57 mice, of untreated *mdx* mice, and of *mdx* mice treated with halofuginone at 7.5 μg/mouse for 10 weeks starting from 4 weeks of age, were immunostained with dystrophin antibodies (red) and collagen type I antibodies (green), nuclei were stained with DAPI (blue) and all were visualized by confocal microscopy. In the diaphragm of the C57/BL mice no collagen was observed and all diaphragm myofibers were stained with dystrophin antibodies. In the *mdx* diaphragm high levels of collagen were observed, together with some RFs. A major decrease in collagen type I levels and increase in the number of RFs were observed after halofuginone treatment.

To investigate the effect of muscle fibrosis on the number of RFs, *mdx* mice were treated with halofuginone at 7.5 μg/mouse, for 10 weeks starting from 4 weeks of age, or with saline, and the diaphragms were immunostained with collagen type I and dystrophin antibodies (Figure 6). In the diaphragms of the WT mice no collagen type I was observed while all myofibers exhibited dystrophin. In the *mdx* mice a major increase in collagen type I levels was observed and only few myofibers exhibited dystrophin. Haofuginone treatment resulted in a decrease in collagen type I levels together with an increase in the number of myofibers exhibiting dystrophin. In the *mdx* diaphragm the RFs were 1.2% of all myofibers, 60% of them were in areas rich in collagen and the rest 40% were in areas rich in utrophin. After halofuginone treatment, the number of RFs increased to 3.4% of all myofibers, 36% of them were in fibrotic areas and the rest 64% were in the utrophin rich area. The numbers of single RFs, i.e., those separated from the nearest neighboring RF by at least two dystrophin-negative fibers, and the numbers of RFs in clusters, i.e., adjacent RFs, in the entire *mdx* diaphragms and gastrocnemius were counted before and after halofuginone treatment (Table 2). In the diaphragm, the number of isolated and clustered RFs was increased after halofuginone treatment while in the gastrocnemius, the major increase was in the number of clustered RFs. The total number of RFs before and after halofuginone treatment was higher in the gastrocnemius than in the diaphragm.

**Table 1:** The effect of halofuginone on RFs number in *mdx* diaphragm and gastrocnemius muscles

Muscle	Treatment	*n*	No. of isolated RFs	No. of clustered RFs	Total no. of RFs
Diaphragm	*mdx*	5	3.8 ± 0.9^a^	3.6 ± 0.5^a^	7.4 ± 1.0^a^
Diaphragm	*mdx* + halo	6	9.1 ± 1.0^b^	12.3 ± 2.6^b^	21.5 ± 3.6^b^
Gastrocnemius	*mdx*	4	11.5 ± 3.2^a^	14.5 ± 4.2^a^	26.0 ± 7.0^a^
Gastrocnemius	*mdx* + halo	5	17.4 ± 1.8^a^	36.2 + 8.2^b^	53.6 ± 6.7^b^

The results are the mean ± SE. Means in the same column and in the same muscle with no common superscript differ significantly (*P* < 0.05).

**Table 2:** DMD patients

Age at Biopsy (y)	Muscle type	Dystrophin	CK U/L	% of utrophin fibers	Clinical data
3	QF	absent	4156	48	Calf hypertrophy, Gowers' sign, difficulty in climbing stairs
4	QF	Few RFs	14000	59	Calf hypertrophy
5	QF	absent	23000	73	Calf hypertrophy, Gowers' sign, waddling gait, proximal weakness
8	QF	absent	14000	70	Gowers' sign, waddling gait
9	QF	absent	5500	73	Unable to raise from floor, tip-toe walking, upper limb weakness
9	QF	absent	9000	61	Calf hypertrophy, waddling gait, Joint contractures, Gowers' sign

CK- creatine kinase, QF - quadriceps femoris.

## DISCUSSION

**Figure 7:**
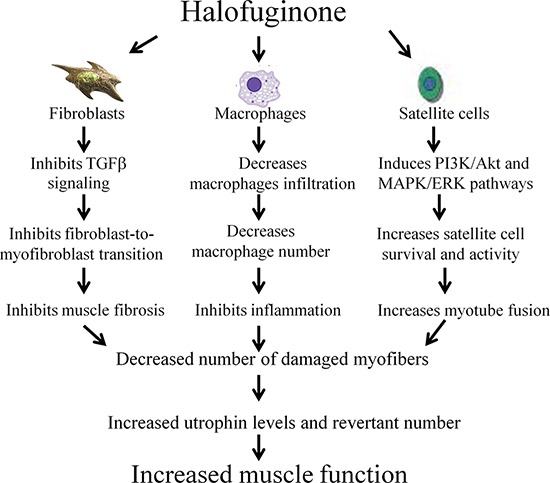
Halofuginone and the dystrophic muscle Halofuginone reduces muscle fibrosis by inhibiting the TGFβ signaling and the fibroblast-to-myofibroblast transition [36–39]. It reduces inflammation by decreasing macrophage infiltration into the dystrophic muscle [37, 38, 42]. Halofuginone promotes myotube fusion and satellite cell survival [41, 58]. All of these halofuginone's actions decrease the number of the damaged myofibers and together with the increase in the utrophin levels and revertant number, they result in increase in muscle function.

The dystrophic muscles of *mdx* mice and of DMD patients are characterized by high levels of fibrosis, on the one hand and, on the other hand, low levels of utrophin and small numbers of RFs that are not sufficient to ameliorate the disease. The level of utrophin in the DMD muscles is around 10 times of that found in normal adult tissue but it is still well below the amount present at the period of maximum expression in the fetus. In DMD, the utrophin-positive fibers corresponded to dystrophin-negative fibers and the majority of muscle fibers are deficient for dystrophin and positive for utrophin [44, 45]. Utrophin level increase with age and it correlates with disease severity as measured by the age at which the patient becomes wheelchair-bound [46]. It was speculated that in DMD muscles the reduction of the amount of utrophin at later age may be partly related to the progress of the disease [47]. Firstly, we demonstrated an inverse correlation between fibrosis and utrophin levels in the dystrophic muscles of both *mdx* mice and DMD patients. This finding is of great importance because identification of common links between the fibrotic reaction and the utrophin-synthesis pathways may lead to new insights into regulation of utrophin synthesis and highlight new targets for pharmaceutical intervention. The underlying principle behind the hypothesized association between utrophin expression and fibrosis arises from indirect evidence: A – In both, *mdx*/utr^−/−^ [14, 22] and *mdx*/utr^+/−^ [48] mice, the fibrosis was more pronounced than in *mdx* mice; B – Fibrosis was reduced in canine X-linked MD by adenovirus-mediated utrophin gene transfer [26]; C – The association between up-regulation of utrophin and decrease in fibrosis has been demonstrated by expression of the four-and-a-half LIM domain protein 1 (FHL1), which significantly increased utrophin levels simultaneously with a decrease in fibrosis in the *mdx* mice [49]. The known actions of halofuginone in MD are summarized in Figure 7.

In the present study, we demonstrated that in DMD patients high levels of collagen type I, the major protein of the fibrotic lesion, were associated with low levels of utrophin and vice versa. This correlation was observed in DMD patients with ages ranging from 3 to 9 years, and it persisted along the entire QF muscle, as demonstrated by double-immunostaining of utrophin and collagen type I of the surface of the biopsies and by z-stack analysis deep into the biopsy (Figures 1, 2). In *mdx* mice, reduction of diaphragm fibrosis by halofuginone treatment was associated with a fivefold increase in utrophin levels (Figure 3). This is of great significance in light of findings that heregulin and arginine butyrate that increased utrophin levels only by 2.8-fold and twofold, respectively, elicited physiological benefits such as improved muscle mechanical properties, as indicated by resistance to eccentric contraction-mediated damage, increased isometric force and decreased CK levels [50, 51]. The levels of utrophin is very low in the diaphragms of old *mdx* mice with established fibrosis and a very high levels of collagen type I. Halofuginone did not affect utrophin synthesis in wild-type mice, which suggests that it probably does not affect utrophin synthesis directly and that the increase in utrophin levels is the result of fibrosis inhibition. The utrophin-fibrosis relationships are not limited to collagen type I but probably apply to all the fibrosis machinery, which is augmented within the dystrophic muscle. The collagen biosynthesis pathway involves the activity of cthrc1, which is up-regulated in the dystrophic muscles of *mdx* mice and of DMD patients, and whose spatial location is close to that of the collagen fibers [33]. Here we show that in the *mdx* diaphragm cthrc1 levels were inversely correlated with utrophin levels also (Figure 4).

The approach of simultaneously up-regulating utrophin expression and reducing fibrosis offers a number of advantages: A – Up-regulation of utrophin in mice had no apparent deleterious consequences [52], and it is not predicted to elicit any immune response in DMD patients – it is already expressed in their muscles; B – No special delivery system is required; C – It will be beneficial to all DMD patients irrespective of mutations; D – Some compounds, although they increased utrophin mRNA, did not elicit increased protein levels [53]. This was probably because of the post-translational and not the post-transcriptional regulatory mechanisms acting on utrophin [15, 54]; E – Inhibition of fibrosis can be used in combination with other modalities to increase utrophin levels in order to achieve even higher utrophin levels in the dystrophic muscles. This is important because recovery from the dystrophic process through over-expression of utrophin is a dose-dependent phenomenon.

Another feature of the dystrophic muscle of the DMD patient is the existence of RFs. The diaphragm, which was the most fibrotic muscle of the *mdx* mice [36], had fewer RFs than any other skeletal muscle [11] and a strong negative correlation was observed between dystrophin expression and the severity of fibrosis in the *mdx/utrn*^−/−^/*Xist*^Δhs^ mice expressing various levels of dystrophin [55]. Inhibition of fibrosis resulted in increased numbers of RFs both in the diaphragm and the gastrocnemius (Table 2). It is interesting to note that various mutant alleles of the *mdx* mice expressed differing numbers of RFs [56]. Although gross histopathology appeared to be similar among the various mutants, the hydroxyproline levels that represent the muscle collagen content and fibrosis differed among a few of them [57]. Thus, the improvement of muscle functions observed in both young and old *mdx* mice after anti-fibrosis treatment [36, 39] was probably due to the combined effects of reduced fibrosis, increased utrophin levels, and increased numbers of RFs.

The results of this study could be explained by at least two mechanisms: A- A general mechanism, independent of the type of the anti-fibrotic agent. In the fibrotic muscle, the excessive deposition of ECM components substitutes the skeletal myofibers with nonfunctional fibrotic tissue, and causes an increase in the number of atrophic myofibers [58], that can no longer express utrophin or dystrophin. Any anti-fibrotic agent that will restore muscle histopathology will increase the total number of viable myofibers, some of which will become dystrophin or utrophin positive. B- Enhanced Akt signaling in the myofibers improves muscle function in the *mdx* mice [59] and promotes the increased expression of utrophin [60]. Halofuginone, that enhances Akt phosphorylation in muscle cells [41, 61] may directly up-regulate utrophin levels in addition to its anti-fibrotic action.

In summary: because the existing strategies to cure MDs, such as gene and cell-replacement therapies have met with some difficulties, the development of complementary and supportive therapies that slow disease progression and improve patients' quality of life is critically important. Thus, a more realistic goal for the near future would be to treat the main pathological mechanisms, e.g., by inhibiting muscle fibrosis and simultaneously increasing utrophin levels and RF numbers in the dystrophic muscle. Such treatments may enable delaying the progressive loss of muscle strength, and improve muscle integrity and function.

## MATERIALS AND METHODS

### Materials

Halofuginone bromhydrate was obtained from Akashi Therapeutics, Inc. (Cambridge, MA, USA); polyclonal mouse-specific collagen type I, monoclonal cthrc1, and dystrophin antibodies were from Abcam (Cambridge, UK); and polyclonal utrophin and polyclonal YY1 antibodies were from Santa Cruz Biotechnology, Inc (Heidelberg, Germany). Monoclonal anti-myosin antibodies for slow and fast fibers were from Sigma-Aldrich (St. Louis, MO, USA). Goat anti-mouse IgG antibodies with Alexa Fluor dye (Molecular Probes, Carlsbad, CA, USA) and Cy3 anti-goat antibodies (Jackson ImmunoResearch Laboratories, Inc., West Grove, PA, USA) were used as secondary antibodies. For western blot analysis, utrophin monoclonal antibodies (BD Biosciences, MD, USA) were used.

### Animals and DMD biopsies

All animal experiments were performed in accordance with the guidelines of the Volcani Center Institutional Committee for Care and Use of Laboratory Animals (IL-433/13). The C57/Bl/6 (wild-type) and *mdx* mice were housed in cages under conditions of constant photoperiod (12/12 h light/dark) with free access to food and water. The mice were injected intra-peritoneally (I.P) with halofuginone at 7.5 μg per mouse, 3 times a week for 10 weeks starting from the age of 4 weeks; the control mice were injected with saline. At the end of the experiments diaphragm and gastrocnemius biopsies were collected for histopathology and western blot analysis [33]. We thank Eurobiobank and Telethon network of Genetic Biobank, Grant number GUP 12007 for the human biopsies. Quadriceps femoris (QF) biopsies of DMD patients who exhibited lack of dystrophin were from the muscle biopsy bank at the Neuromuscular Centre, University of Padova, Italy (Table 1).

### Preparation of sections and immunohistochemistry

Cryo-sections were immunostained with utrophin (1:50) alone, or double-immunostained with collagen type I (1:100) and cthrc1 (1:50) antibodies. Cell nuclei were stained with DAPI (1:1000). As secondary antibodies goat anti-mouse IgG antibodies with Alexa Fluor dye were used for collagen type I detection and Cy3 anti-goat for utrophin detection. Microscope observations and image acquisition were performed with the Olympus IX 81 inverted laser scanning confocal microscope (Fluoview 500; Olympus, Tokyo, Japan), equipped with a 40 -nm diode laser, a 488-nm argon-ion laser, a 543-nm helium-neon laser, and a 60 × 1.0 NA PlanApo water-immersion objective. The transmitted-light images were obtained by means of Nomarski differential interference contrast. The Z-stacks were each 1.2 μm each and extended 8 μm into the tissue. “GREEN” was excited at 488 nm and the emission was collected through a BA 505–525 filter; “RED” was excited at 543 nm and the emission was collected through a BA 560 IF filter. The 3-D reconstruction of the utrophin, collagen type I and cthrc1 levels was prepared by Nis-Elements AR software version 3.2 (Nikon Instruments Inc., Melville, NY, USA), and their levels were evaluated by NIS-Elements microscope imaging software (Nikon Instruments Inc).

### Revertant fiber evaluation

Diaphragm biopsies were immunostained with dystrophin antibodies. Fibers were counted as revertants only when the entire membrane circumference in the cross-sections was stained. RFs adjacent to each other were considered as part of a cluster and were regarded as single RFs when separated by at least two dystrophin-negative fibers from the nearest neighboring RF [8, 11]. Total number of RFs, the number of isolated RFs and numbers of RFs in clusters were recorded and represented as means per diaphragm of at least five mice.

### Western blot analysis

Western blot analysis was performed according to Roffe et al. [41]. Briefly: equal amounts of protein, from a pool of three diaphragms from each group were resolved by 10% SDS-PAGE and then transferred to nitrocellulose membranes (Bio-Rad Laboratories, Hercules, CA, USA). After blocking, the membranes were incubated with mouse utrophin antibodies (1:50) and rabbit polyclonal YY1 (1:500) antibodies. The transcriptional repressor protein YY1 was chosen because its gene expression in diaphragms of *mdx* mice did not differ from that in diaphragms of the C57/BL mice [33].

### Statistical analysis

The data were subjected to one-way analysis of variance and to the all-pairs Tukey-Kramer honestly significant difference test by using JMP Jump Pro-Statistical Discovery software (SAS, Cary, NC, USA).
